# *VEGF-C* Gene Polymorphisms Increase Susceptibility to Rheumatoid Arthritis

**DOI:** 10.7150/ijms.34659

**Published:** 2019-09-20

**Authors:** Chengqian Dai, Shu-Jui Kuo, Sung-Lin Hu, Chun-Hao Tsai, Yuan-Li Huang, Chien-Chung Huang, Lihong Wang, Guohong Xu, Chen-Ming Su, Chih-Hsin Tang

**Affiliations:** 1 Department of Orthopedics, Affiliated Dongyang Hospital of Wenzhou Medical University, Dongyang, Zhejiang, China; 2 School of Medicine, China Medical University, Taichung, Taiwan; 3 Department of Orthopedic Surgery, China Medical University Hospital, Taichung, Taiwan; 4 Department of Family Medicine, China Medical University Hsinchu Hospital, Hsinchu, Taiwan; 5 Department of Biotechnology, College of Health Science, Asia University, Taichung, Taiwan; 6 Division of Immunology and Rheumatology, Department of Internal Medicine, China Medical University Hospital, Taichung, Taiwan; 7 Department of Biomedical Sciences Laboratory, Affiliated Dongyang Hospital of Wenzhou Medical University, Dongyang, Zhejiang, China; 8 Chinese Medicine Research Center, China Medical University, Taichung, Taiwan

**Keywords:** single nucleotide polymorphism, rheumatoid arthritis, VEGF-C

## Abstract

Vascular endothelial growth factor C (VEGF-C) promotes angiogenesis, a prominent feature in rheumatoid synovitis, contributing to the perpetuation of the global burden of rheumatoid arthritis (RA). *VEGF-C* gene polymorphisms predict the risk of developing various human diseases, such as urothelial cell carcinoma, oral cancer and coronary artery disease. We sought to determine whether single nucleotide polymorphisms (SNPs) of the *VEGF-C* gene can predict the risk of RA. Our study recruited 210 patients with RA and 373 healthy controls between 2007 and 2015, and performed comparative genotyping for SNPs rs7664413, rs11947611, rs1485766, rs2046463 and rs3775194. In analyses adjusted for potential covariates, we found that compared with subjects with the A/A genotype of SNP rs11947611, those with the A/G genotype were 40% more likely to develop RA (adjusted odds ratio [AOR] 0.61; 95% confidence interval [CI] 0.40 to 0.92; *p* = 0.02). In addition, subjects lacking the A/A genotype (A/G, G/G) of SNP rs2046463 were more than twice as likely as those with the A/A genotype to require methotrexate (AOR 2.23, 95% CI 1.25 to 3.98; *p* = 0.01), while those who lacked the G/G genotype (G/C, C/C) in the SNP rs3775194 had a significantly lower risk of requiring prednisolone as compared with those with the G/G genotype (AOR 0.39, 95% CI 0.19 to 0.79; *p* = 0.01). Our findings suggest that *VEGF-C* gene polymorphisms might serve as a diagnostic marker and therapeutic target for RA therapy. Pharmacotherapies that modulate the activity of the *VEGF-C* gene may be promising for RA treatment.

## Introduction

Rheumatoid arthritis (RA) is an autoimmune disease that manifests hypertrophy and hypervascularity in synovial tissues and leads to joint destruction, affecting approximately 1% of the global population 1-4. Despite several treatment regimens emerging in recent years that have enabled a substantial portion of RA patients to achieve disease remission with minimal symptoms, some patients remain treatment-refractory and continue to experience progressive joint deterioration and increasing functional limitations, or even premature mortality 5-7. When compared with the general population, RA patients face higher risks of major morbidities, including infection and pulmonary and renal disease, and an approximate 1.5-fold higher risk of mortality 8. The fact that genetic factors account for around 60% of the overall susceptibility to RA highlights the importance of research into the genetic basis for RA 5, 9. Research into RA genetics may facilitate risk prediction and enable individually tailored treatment 6, 10.

A single nucleotide polymorphism (SNP) is a variation in a single nucleotide occurring at a specific site in the genome 11-14. Comparisons of SNP distribution frequencies among patient populations (e.g., patients and controls) are commonly applied to predict disease risk and prognosis, including RA 15, 16. For instance, polymorphisms of the vascular endothelial growth factor C (*VEGF-C*) gene can predict the risk and prognosis of various diseases, including urothelial cell carcinoma, oral cancer, and coronary artery disease 17-19.

VEGFs are pivotal regulators of angiogenesis and include 5 members in mammals: VEGF (or VEGF-A), placental growth factor, VEGF-B, VEGF-C and VEGF-D 19. The VEGF-A signaling cascade via VEGF receptor-2 is considered to be the main angiogenic pathway 20, 21. VEGF receptor-1 inhibits VEGF-mediated angiogenesis during development but enhances pathological angiogenesis when activated by placental growth factor and VEGF-B 22-24. Both VEGF-C and VEGF-D facilitate lymphangiogenesis via VEGF receptor-3, although VEGF-C is the major player in both physiological and pathological lymphangiogenesis 25-27.

The correlation between VEGF-C and RA pathogenesis has been discussed previously 28. Increased VEGF-C expression has been observed in the synovial lining of RA patients 29 and levels of VEGF-C expression reflect the severity of synovitis in early RA 30. However, despite the recognized impact of VEGF-C on RA pathogenesis and the known prognostic value of *VEGF-C* SNPs for human disease, little is known about the potential association between *VEGF-C* SNPs and the risk of RA. In this study, we sought to determine the predictive capacity of *VEGF-C* SNPs as potential biomarkers for RA susceptibility.

## Materials and Methods

### Patients and blood samples

We collected whole blood samples (3 mL) from 210 patients diagnosed with RA at Dongyang People's Hospital, Zhejiang Province, China, and from 373 healthy voluntary donors without RA or any history of malignancy (control group) between 2007 and 2015. All the study participants provided written informed consent for the study, which was approved by the Dongyang People's Hospital Ethics Committee and Institutional Review Board (2015-YB002). Clinical and pathological characteristics of all participants were obtained from medical records, including age at disease onset and blood sampling data, gender, and treatment regimens. At baseline, serum samples were collected from all RA patients and analyzed for levels of anti-citrullinated protein antibodies (ACPAs), rheumatoid factor (RF), erythrocyte sedimentation rate (ESR) and C-reactive protein (CRP). Samples were deemed ACPA-positive if serum titers of anti-cyclic citrullinated peptide antibodies (anti-CCP2) were ≥17 IU/mL and RF-positive if immunoglobulin M (IgM) RF titers were ≥30 IU/mL. All blood samples were stored at -80°C for subsequent analysis.

### Selection of *VEGF-C* polymorphisms

Five *VEGF-C* SNPs were selected from the intron of the *VEGF-C* gene. All SNPs had minor allele frequencies of greater than 5%.

### Genomic DNA extraction

Genomic DNA was extracted from leukocytes in the peripheral blood utilizing a QIAamp DNA blood kit (Qiagen, CA, USA), following the manufacturer's instructions. Extracted DNA was stored at -20°C and prepared for genotyping by polymerase chain reaction (PCR) 31, 32.

### Genotyping by real-time PCR

Five VEGF-C SNP probes were purchased from Applied Biosystems (CA, USA). The recognition of allelic discrimination for *VEGF-C* SNPs was conducted utilizing a QuantStudioTM 5 Real-Time PCR system, following the manufacturer's instructions. Data were analyzed with QuantStudio^™^ Design and Analysis Software (Applied Biosystems, CA, USA). PCR was performed in a total volume of 10 μL, containing 20-70 ng genomic DNA, 1 U Taqman Genotyping Master Mix, and 0.25 μL probes. The PCR protocol included an initial 10-min denaturation step at 95°C, followed by 40 cycles of 95°C for 15 s and 60°C for 1 min 33, 34.

### Statistical analysis

Between-group differences were considered significant if *p* values were less than 0.05. Hardy-Weinberg equilibrium (HWE) was assessed using Chi-square goodness-of-fit tests for biallelic markers. Fisher's exact test was utilized to compare differences in demographic characteristics between the two groups. The odds ratios (ORs) and 95% confidence intervals (CIs) for associations between genotype frequencies and the risk of RA or pertinent characteristics were estimated by multiple logistic regression models that controlled for age and gender. All data were analyzed using Statistical Analytic System software (v. 9.1, 2005; SAS Institute, Cary, NC, USA).

## Results

All of the study participants were of Chinese Han ethnicity. The mean age at blood sampling was 55.05 ± 11.75 years for the RA cohort and 42.08 ± 19.02 years for the controls (*p* < 0.001). The proportion of female participants was 82.9% in the RA cohort and 56.6% in the control cohort (*p* < 0.0001). The interval between the onset of RA and the blood sampling was 53.73 ± 68.58 months. At the time of blood sampling, 60.0% of the RA cohort were receiving TNF-α inhibitors, 43.3% were receiving methotrexate, and 45.7% were receiving prednisolone. The majority of RA patients were rheumatoid factor (RF)-positive (85.2%) and anti-citrullinated protein antibody (ACPA)-positive (78.6%) (Table 1).

Polymorphism frequencies for both cohorts are detailed in Table 2. All genotypes were in Hardy-Weinberg equilibrium (*p* > 0.05). The most frequent genotypes for SNPs rs7664413, rs11947611, rs1485766. rs2046463 and rs3775194 were C/C, A/G, G/T, A/A and G/G for the RA cohort and C/C, A/A, G/T, A/G, and G/G for the controls, respectively. Having the A/G genotype of SNP rs11947611 significantly decreased the risk of developing RA disease, compared with having the A/A genotype (adjusted OR [AOR] 0.61; 95% CI, 0.40 to 0.92; *p* = 0.02).

RA disease occurred at the age of <35 years in 25 patients and at age ≥35 years in 185 patients. Analyses of the data for any correlation between SNPs and RA onset before 35 years of age revealed that having the SNP rs7664413 C/T genotype was associated with an ~80% lower likelihood of RA compared with having the wild-type C/C genotype (AOR 0.21; 95% CI, 0.06 to 0.78; *p* = 0.02), which was also the case for patients who had the SNP rs2046463 A/G genotype compared with those who had the wild-type A/A genotype (AOR 0.20; 95% CI, 0.05 to 0.74; *p* = 0.02), while those who had the SNP rs11947611 non-A/A genotype (A/G, G/G) had a ~3-fold higher risk of developing RA compared with those who had the A/A genotype (AOR 3.23; 95% CI, 1.04 to 10.06; *p* = 0.04) (Table 3).

Analyses failed to find any correlations between serum levels of RA biomarkers (RF, ACPA, ESR and CRP) and the SNP rs11947611 at the time of blood sampling (Table 4). In an analysis of SNPs and medication requirements, patients who had the non-C/C (C/T, T/T) genotype of the SNP rs7664413 had a higher than 2-fold risk of requiring methotrexate as compared with patients who had the C/C genotype (AOR 2.07; 95% CI, 1.17 to 3.68; *p* = 0.01), as did those who had the non-A/A (A/G, G/G) genotype of the rs2046463 SNP as compared with patients who had the A/A genotype (AOR 2.23; 95% CI, 1.25 to 3.98; *p* = 0.01), while patients who had the non-G/G (G/C, C/C) genotype of the rs3775194 SNP had a significantly reduced risk of requiring steroids compared with patients who had the G/G genotype (AOR 0.39; 95% CI, 0.19 to 0.79; *p* = 0.01) (Table 5).

## Discussion

Although the advent of biological-based therapies has helped many patients with RA to achieve good disease control, some remain treatment-refractory 35. It is therefore critical that investigations continue to explore the pathogenesis of RA. One promising avenue of research is the identification of RA-related SNPs, which will also enable risk stratification of patients for treatment 36.

*VEGF-C* SNPs have prognostic value in various diseases 17-19. A Taiwanese study has reported that the *VEGF-C* rs1485766 polymorphism appears to contribute to the risk of urothelial cell carcinoma (UCC) 17 . That study also found that smokers with certain genetic variants of *VEGF-C* were more likely to develop UCC and larger-sized tumors than smokers with wild-type homozygotes. Another Taiwanese study has reported that *VEGF-C* rs7664413 and rs2046463 polymorphisms and either of 2 haplotypes of 5 *VEGF-C* combined were related to the risk of oral cancer 18. The study researchers also found that among male smokers, *VEGF-C* variant carriers who also chewed betel quid were 14-5-24.2-fold more likely to have oral cancer compared to *VEGF-C* wild-type carriers who did not chew betel quid. Conversely, in the cohort of men who chewed betel quid, those carrying the *VEGF-C* polymorphism who also smoked were 2.7-18.2-fold to have oral cancer compared to those who carried the wild type but did not smoke. Interestingly, Japanese research has found that having a low *VEGF-C* level is significantly and inversely associated with all-cause mortality among patients with suspected or known coronary artery disease, which contrasts with the fact that high *VEGF-C* levels are apparently associated with poor prognosis in patients with malignancies 19.

Despite the evidence inferring a role for *VEGF-C* in the pathogenesis of RA and the prognostic capacity of *VEGF-C* SNPs in human diseases, few studies have investigated the relationship between *VEGF-C* SNPs and risk of RA development and progression. We therefore sought to determine the prognostic capacity of *VEGF-C* SNPs in predicting RA onset. To the best of our knowledge, our study is the first to identify that the *VEGF-C* rs7664413, rs11947611, rs1485766, rs2046463, and rs3775194 polymorphisms are related to RA development. We found that having the A/G genotype of SNP rs11947611 exerted a significantly protective effect against developing RA (*p* = 0.02).

When we analyzed associations between the 5 *VEGF-C* SNPs and age of RA onset, we found a significantly protective effect for the heterozygous genotype (C/T) of SNP rs7664413 for the development of RA disease before 35 years of age, which was also the case for patients who had the A/G genotype of rs2046463 (*p* = 0.02 for each comparison with the wild-type genotypes of each polymorphism). Conversely, RA patients who had the non-A/A genotype (A/G, G/G) of SNP rs11947611 had a substantially higher risk of developing RA before the age of 35 years compared with their wild-type counterparts (*p* = 0.04). In analyses of requirements for RA medications, patients with the non-C/C genotype (C/T, T/T) of rs7664413 were significantly more likely to require methotrexate, as were those with the non-A/A genotype (A/G, G/G) of rs2046463 (*p* = 0.01 for both comparisons vs the wild-type genotypes of each polymorphism, respectively), while RA patients who had the non-G/G genotype (G/C, C/C) of rs3775194 were significantly less likely to require steroidal treatment as compared with their wild-type counterparts (*p* = 0.01). Further explorations are warranted into correlations between clinical profiles of RA disease activity and genetic variants of the *VEGF-C* gene.

A major limitation of our study is that our findings might merely reflect a cross-sectional relationship instead of actual causality. This is a common limitation of similar studies and could potentially be overcome by more in-depth investigations analyzing the relationships between all known SNP elements. Besides, some important clinical parameters are not ubiquitously available from the existing medical records, such as disease activity score -28 (DAS-28), Larsen score, and visual analogue scale (VAS). More comprehensive documentation of clinical parameters should be warranted in the future studies.

In conclusion, our study offers novel insights into *VEGF-C* SNPs in regard to RA susceptibility. We found that A/G genotype of SNP rs11947611 was a protective factor for RA development. This is the first study to show associations between *VEGF-C* polymorphisms and RA susceptibility, early age of onset and treatment regimens. Thus, *VEGF-C* appears to be a diagnostic marker and therapeutic target for RA therapy; therapeutic agents that directly or indirectly regulate *VEGF-C* activity may prove promising in the treatment of RA disease.

## Figures and Tables

**Table 1:** Demographic characteristics of the study population.

Variables		Healthy controls (n=373)	RA patients (n=210)	OR	(95% CI)	*p* value
		N (%)	N (%)			
Age (years) (mean ± SD)		42.08 ± 19.02	55.05 ± 11.75			**< 0.0001**
**Sex**						
Female		211 (56.6)	174 (82.9)	1		
Male		162 (43.4)	36 (17.1)	0.27 (0.18 to 0.41)		**< 0.0001**
**RF**						
seropositive	-	ND	31 (14.8)			
	+	ND	179 (85.2)			
**ACPA**						
	-	ND	45 (21.4)			
	+	ND	165 (78.6)			
**Anti-TNF**						
	-	ND	85 (40.0)			
	+	ND	126 (60.0)			
**Methotrexate**						
	-	ND	119 (56.7)			
	+	ND	91 (43.3)			
**Prednisolone**						
	-	ND	114 (54.3)			
	+	ND	96 (45.7)			
**ESR (mean ± SD)**			30.54 ± 24.82			
< 20		ND	96 (45.7)			
≥ 20		ND	114 (54.3)			
**CRP(mean ± SD)**			22.68 ± 82.31			
< 8		ND	128 (61.0)			
≥ 8		ND	82 (39.0)			
RA duration, months (mean ± SD)		ND	53.73 ±68.58			

RA = rheumatoid arthritis; OR= odds ratio; CI = confidence interval; RF = rheumatoid factor; ACPA = anti-citrullinated protein antibodies; ND = not determined; ESR = erythrocyte sedimentation rate; CRP = C-reactive protein.

**Table 2:** VEGF-C genotyping frequencies in cases and controls and the association with risk of RA.

Variables	Control(n=373)	Case(n=210)	OR	(95% CI)	*P* Value	AOR^a^	(95% CI)	*P* Value	HWE^b^
	N (%)	N (%)							
**rs7664413**									0.03/0.98
CC	182 (48.8)	98 (46.7)	1			1			
CT	141 (37.8)	92 (43.8)	1.21	(0.85-1.74)	0.29	1.25	(0.83-1.90)	0.25	
TT	50 (13.4)	20 (9.5)	0.74	(0.42-1.32)	0.31	0.56	(0.29-1.07)	0.08	
CT+TT	191 (51.2)	112 (53.33)	1.09	(0.78-1.53)	0.62	1.04	(0.71-1.53)	0.84	
**rs11947611**									0.95/0.10
AA	161 (43.2)	110 (52.4)	1			1			
AG	166 (44.5)	75 (35.7)	**0.66**	**(0.46-0.95)**	**0.03**	**0.61**	**(0.40-0.92)**	**0.02**	
GG	46 (12.3)	25 (11.9)	0.8	(0.46-1.37)	0.41	0.74	(040-1.39)	0.34	
AG+GG	212 (56.8)	100 (47.6)	0.69	(0.49-0.97)	0.03	0.64	(0.43-0.94)	0.03	
**rs1485766**									0.47/0.06
GG	111 (29.8)	47 (22.4)	1			1			
GT	174 (46.6)	104 (49.5)	1.12	(0.76-1.68)	0.56	0.98	(0.62-1.55)	0.93	
TT	88 (23.6)	59 (28.1)	1.01	(0.63-1.62)	0.98	1.06	(0.62-1.81)	0.86	
GT+TT	262 (70.24)	151 (71.9)	1.08	(0.75-1.58)	0.67	1.01	(0.66-1.54)	0.98	
**rs2046463**									0.03/0.82
AA	181 (48.5)	94 (44.8)	1			1			
AG	141 (37.8)	96 (45.7)	1.31	(0.92-1.88)	0.34	1.35	(0.89-2.05)	0.15	
GG	51 (13.7)	20 (9.5)	0.76	(0.43-1.34)	0.14	0.57	(0.30-1.10)	0.09	
AG+GG	192 (51.5)	116 (55.2)	1.16	(0.83-1.63)	0.38	1.11	(0.76-1.64)	0.59	
**rs3775194**									0.99/0.97
GG	311 (83.4)	168 (80.0)	1			1			
CG	59 (15.8)	40 (19.0)	1.26	(0.81-1.96)	0.32	1.38	(0.82-2.32)	0.22	
CC	3 (0.8)	2 (1.0)	1.23	(0.20-7.46)	0.82	1.17	(0.17-7.96)	0.87	
CG+CC	62 (16.6)	42 (20.0)	1.25	(0.81-1.94)	0.31	1.37	(0.83-2.27)	0.22	

^a^ Logistic regression with adjustment for age, gender. ^b^ Control/Case Hardy-Weinberg equilibrium test.

**Table 3:** The association with VEGF-C genotyping frequencies and age onset of RA.

	Age-onset < 35 years		OR (95 % CI)	*p* Value	AOR^a^ (95% CI)	*p* Value
	(n=185) N(%)	(n=25) N(%)				
Age, year (mean±SD)	39.28± 11.77	57.18 ± 10.02		< 0.0001		
**rs7664413**						
CC	82 (44.3)	16 (64.0)	1		1	
CT	85 (46.0)	7 (28.0)	**0.42 (0.17-1.08)**	**0.07**	**0.21(0.06-.078)**	**0.02**
TT	18 (9.7)	2 (8.0)	0.57 (0.12-2.70)	0.48	0.4(0.06-2.67)	0.34
CT+TT	103 (55.7)	9 (36.0)	**0.45(0.19-1.07)**	**0.07**	**0.24(0.07-0.80)**	**0.02**
**rs11947611**						
AA	101 (54.6)	9 (36.0)	1		1	
AG	64 (34.6)	11 (44.0)	**1.93(0.76-4.91)**	**0.17**	**3.26(0.96-11.07)**	**0.06**
GG	20 (10.8)	5 (20.0)	2.81(0.85-9.26)	0.09	3.16(0.61-16.46)	0.17
AG+GG	84 (45.4)	16 (64.0)	**2.14(0.90-5.08)**	**0.09**	**3.23(1.04-10.06)**	**0.04**
**rs1485766**						
GG	55 (29.7)	4 (16.0)	1		1	
GT	93 (50.3)	11 (44.0)	1.63(0.49-5.36)	0.42	2.28(0.53-9.82)	0.27
TT	37 (20.0)	10 (40.0)	3.72(1.08-12.70)	0.04	3.67(0.80-16.96)	0.09
GT+TT	130 (70.3)	151 (71.9)	2.22(0.73-6.77)	0.16	2.79(0.71-10.93)	0.14
**rs2046463**						
AA	78 (42.2)	16 (64.0)	1		1	
AG	89 (48.1)	7 (28.0)	0.38(0.15-0.98)	0.04	0.2(0.05-0.74)	0.02
GG	18 (9.7)	2 (8.0)	0.54(0.11-0.57)	0.44	0.38(0.05-0.60)	0.33
AG+GG	107 (57.8)	9 (36.0)	0.41(0.17-0.98)	0.04	0.23(0.07-0.76)	0.02
**rs3775194**						
GG	146 (78.9)	22 (88.0)	1		1	
CG	37 (20.0)	3 (12.0)	0.54(0.15-1.89)	0.33	0.44(0.09-2.18)	0.32
CC	2 (1.1)	0 (0.0)				
CG+CC	39 (21.1)	2 (12.0)	0.51(0.15-1.79)	0.29	0.39	0.24

^a^ Logistic regression with adjustment for age, gender.

**Table 4:** Clinical biochemical status and VEGF‐C rs11947611 genotypic frequencies in RA Patients*

Variables		AA (n=110)	AG+GG (n=100)	OR (95% CI)	*p* value	AOR^a^(95% CI)	*p* value
		N (%)	N (%)				
**rs11947611**							
**RF**	Negative	16 (14.5)	15 (15.0)	1		1	
	Positive	94 (85.5)	85 (85.0)	0.97(0.45-2.07)	0.93	0.97(0.45-2.09)	0.94
**ACPA**	Negative	25 (22.7)	20 (20.0)	1			
	Positive	85 (77.3)	80 (80.0)	1.18(0.61-2.28)	0.63	1.15(0.59-2.25)	0.67
**ESR**	< 20	50 (45.5)	46 (46.0)	1		1	
	≥ 20	60 (54.5)	54 (54.0)	0.98(0.57-1.69)	0.94	0.97(0.56-1.68)	0.92
**CRP**	< 8	65 (59.1)	63 (63.0)	1		1	
	≥ 8	45 (40.9)	37 (37.0)	0.85(0.49-1.48)	0.56	0.86(0.49-1.51)	0.6

*RF= rheumatoid factor; ACPA= anti-citrullinated protein antibodies; ESR= erythrocyte sedimentation rate; CRP= C-reactive protein; ^a^ Logistic regression with adjustment for age, gender.

**Table 5:** The association with VEGF-C genotyping frequencies and RA medication.

Variables		N (%)	N (%)	OR (95% CI)	*P* Value	AORa (95% CI)	*P* Value
**rs7664413**		**CC (n=98)**	**CT+TT (n=112)**				
Anti-TNF	-	53 (54.1)	73 (65.2)	1		1	
	+	45 (45.9)	39 (34.8)	0.63(0.36-1.10)	0.1	0.65(0.37-1.14)	0.13
Methotrexate	-	51 (52.0)	40 (35.7)	1		1	
	+	47 (48.0)	72 (64.3)	**1.95(1.12-3.40)**	**0.02**	**2.07(1.17-3.68)**	**0.01**
Prednisolone	-	49 (50.0)	47 (42.0)	1		1	
	+	49 (50.0)	65 (58.0)	1.38(0.80-2.34)	0.24	1.36(0.78-2.35)	0.28
**rs2046463**		**AA (n=94)**	**AG+GG (n=116)**				
Anti-TNF	-	50 (53.2)	76 (65.5)	1		1	
	+	44 (46.8)	40 (34.5)	0.60(0.34-1.04)	0.07	0.62(0.36-1.10)	0.1
Methotrexate	-	50 (53.2)	41 (35.3)	1		1	
	+	44 (46.8)	75 (64.7)	**2.08(1.19-3.62)**	**0.01**	**2.23(1.25-3.98)**	**0.01**
Prednisolone	-	48 (51.1)	48 (41.4)	1		1	
	+	46 (48.9)	68 (58.6)	1.48(0.86-2.56)	0.16	1.44(0.83-2.49)	0.2
**rs3775194**		**GG (n=168)**	**GC+CC (n=42)**				
Anti-TNF	-	102 (60.7)	24 (57.1)	1		1	
	+	66 (39.3)	18 (42.9)	1.15(0.58-2.30)	0.67	1.13(0.57-2.27)	0.73
Methotrexate	-	74 (44.1)	17 (40.5)	1		1	
	+	94 (55.9)	25 (59.5)	1.16(0.57-2.30)	0.68	1.27(0.62-2.59)	0.51
Prednisolone	-	69 (41.1)	27 (64.3)	1		1	
	+	99 (58.9)	15 (35.7)	**0.39(0.19-0.78)**	**0.01**	**0.39(0.19-0.79)**	**0.01**

^a^ Logistic regression with adjustment for age, gender.
